# Investigating Effects of Plasma Apolipoprotein E on Ischemic Heart Disease Using Mendelian Randomization Study

**DOI:** 10.3390/nu13072215

**Published:** 2021-06-28

**Authors:** Meng-Yu Li, Man-Ki Kwok, Catherine Mary Schooling

**Affiliations:** 1 Li Ka Shing Faculty of Medicine, School of Public Health, The University of Hong Kong, Patrick Manson Building (North Wing), 7 Sassoon Road, Pokfulam, Hong Kong, China; u3005164@connect.hku.hk (M.-Y.L.); maggiek@hku.hk (M.-K.K.); 2 Graduate School of Public Health and Health Policy, City University of New York, 55 West 125th Street, New York, NY 10027, USA

**Keywords:** apolipoprotein E, ischemic heart disease, Mendelian Randomization, genetics, lipids

## Abstract

Background: Observationally plasma apolipoprotein E (apoE) is positively associated with ischemic heart disease (IHD). A Mendelian randomization (MR) study suggesting apoE is unrelated to cardiovascular mortality did not consider specific isoforms. We used MR to obtain estimates of plasma apoE2, apoE3 and apoE4 on IHD, low-density lipoprotein (LDL) and high-density lipoprotein (HDL) cholesterol, triglycerides and apolipoprotein B (apoB). Methods: We obtained independent genetic instruments from proteome genome-wide association studies (GWAS) and applied them to large outcome GWAS. We used univariable MR to assess the role of each isoform and multivariable MR to assess direct effects. Results: In univariable MR, apoE4 was positively associated with IHD (odds ratio (OR) 1.05, 95% confidence interval (CI) 1.01 to 1.09), but apoE2 and apoE3 were less clearly associated. Using multivariable MR an association of apoE2 with IHD (OR 1.16, 95% CI 0.98 to 1.38) could not be excluded, and associations of apoE3 and apoE4 with IHD were not obvious. In univariable MR, apoE2 and apoE4 were positively associated with apoB, and a positive association of apoE2 with LDL cholesterol could not be excluded. Using multivariable MR apoE2 was positively associated with LDL cholesterol, and associations with apoB could not be excluded. After adjusting for apoB, no direct effects of apoE isoforms on IHD were evident. Conclusions: Plasma apoE2 and apoE4 may play a role in lipid modulation and IHD. Whether apoE could be a potential therapeutic target requires further clarification when larger genetic studies of apoE isoforms are available.

## 1. Background

Apolipoprotein E (apoE) is known for its role in lipid transport and regulation as a ligand to the low-density lipoprotein (LDL) receptor [1]. *APOE* genetic variants also affect apolipoprotein B (apoB) [2,3], whose relevance to ischemic heart disease (IHD) is increasingly acknowledged [4,5,6,7]. Correspondingly, apoE is also emerging as an overlooked target [8], which might be modulated by statins [9,10,11]. ApoE has three (apoE2, apoE3 and apoE4) isoforms, which differ by one or two amino acids but are functionally different [12]. The different apoE isoforms are coded by three haplotypes (ε2, ε3 and ε4). ε3 (rs429358-T, rs7412-C) allele is the most common allele [13]. ε2 (rs429358-T, rs7412-T) is associated with lower risk of IHD [14,15,16,17,18], lower LDL cholesterol [14] and lower plasma apolipoprotein B (apoB) [2,19,20,21], compared to ε3ε3. A recent study showed that ε2ε2 genotype was positively associated with cardiovascular conditions including peripheral vascular disease and thromboembolism [22]. In comparison, ε4 (rs429358-C, rs7412-C) is associated with a higher risk of IHD [14,15,16,17,18,23], higher LDL cholesterol [14] and higher plasma apoB [2,19,20,21] compared to ε3ε3. ApoE3 is the parent isoform, associated with normal plasma lipids [24]. ApoE2 has the lowest affinity for the LDL receptor, and has less of a role in lipid clearance, so is associated with type III hyperlipoproteinemia [24], a risk factor for atherosclerosis [25]. ApoE4 has a preference for very-low-density lipoprotein (VLDL), and is associated with a pro-atherogenic lipid profile (high VLDL cholesterol/high-density lipoprotein (HDL) cholesterol ratio) [24]. Observationally, plasma apoE protein is positively associated with IHD and myocardial infarction in humans [26], but is anti-atherosclerotic in mice [27]. Overall conflicting evidence and opinions have made the effects of plasma apoE mysterious [27], with a dearth of experimental evidence in humans.

Although the main two *APOE* coding variants result in qualitative differences in apoE isoforms, *APOE* genetic variants are pleiotropic [22], making it difficult to infer the target of intervention, or inform the functionality of apoE proteins. In these circumstances Mendelian randomization (MR) offers a way forward because MR uses genetic proxies to obtain less confounded estimates. The random allocation of genetic variants at conception obviates confounding [28]. A previous MR study found no association of apoE with cardiovascular disease mortality, but did not consider isoform specific effects [29], and may be open to selection bias given about a 20-year gap between initial recruitment and sampling for genotyping in some participants which could attenuate the estimates. To provide more insight, we conducted both univariable and multivariable MR study to assess the effects of plasma apoE2, apoE3 and apoE4 on IHD and lipid profile, including LDL cholesterol, HDL cholesterol and triglycerides, as well as apoB. For completeness, we also conducted a multivariable MR study to assess the direct effects of these apoE isoforms on IHD after adjusting for apoB to further investigate whether the effects of apoE isoforms are relevant to apoB.

## 2. Materials & Methods

### 2.1. Study Design

This is a two-sample MR study, using genetic summary statistics for exposures and outcomes from the largest available genome-wide association studies (GWAS) [30,31,32,33,34]. Estimates of associations of exposures on outcomes were obtained by meta-analyzing genetic variant-specific Wald estimates (genetic association with outcome divided by association with exposure) using different methods.

### 2.2. Data Sources

#### 2.2.1. Genetic Predictors of Plasma ApoE Isoforms

We selected genetic predictors, i.e., single-nucleotide polymorphisms (SNP), strongly (*p*-value < 10^−5^) and independently (r^2^ < 0.001) predicting plasma apoE2, apoE3 and apoE4 given the two coding variants were not available. Selecting genetic instruments statistically across the genome captures both cis-and trans-variants may also give a more precise and comprehensive proxy of the relevant exposure. Genetic associations with plasma apoE2, apoE3 and apoE4 were obtained from a proteome GWAS in up to 997 participants from the German KORA F4 study, which is a follow-up study of KORA S4 [30]. Genotyping was done using Affymetrix Axiom Array for participants in KORA S4 (mean age 49 years). Blood samples were drawn from participants in the KORA F4 (48.4% male) in the morning after 10+ hours’ overnight fasting, and the protein levels were quantified using the SOMAscan platform, as previously [30,35]. The genetic associations with inversed-normalized probe levels were adjusted for age, gender and body mass index [30]. As a validation for univariable associations with apoE2 and apoE3, we selected genetic predictors strongly (*p*-value < 5 × 10^−8^) and independently (r^2^ < 0.05) predicting plasma apoE2 and apoE3 (standard deviation) from a larger proteome GWAS from the INTERVAL study, which was conducted in 3301 participants (mean age 43.7 years, 51.1% male) of European ancestry, adjusted for age, sex, duration between blood draw and processing (≤1 day/>1 day) and principal components of ancestry [31]. The “ld_clump” function in the ieugwasr R package was used to select the independent genetic variants. Validation for apoE4 could not be done because genetic associations with apoE4 are not available in the INTERVAL study.

Proxies (r^2^ > 0.6), obtained from LD Link (https://ldlink.nci.nih.gov/?tab=ldproxy (accessed on 2 May 2019)), were used for palindromic SNPs (A/T or G/C) and for SNPs not available for an outcome. No proxies were used for genetic variants from the KORA study since all genetic variants were available for both exposure and outcome and were not palindromic When using genetic variants from the INTERVAL study, the outcome information for rs814573 was replaced by that for rs4420638 (r^2^ = 0.88) for both apoE2 and apoE3 on all outcomes, and for rs1065853 was replaced by rs7412 (r^2^ = 1.0) for apoE2 and apoE3 on IHD and apoB.

#### 2.2.2. Genetic Associations with IHD, LDL Cholesterol, HDL Cholesterol, Triglycerides and ApoB

Genetic associations with IHD were obtained from publicly available GWAS summary statistics, i.e., the CARDIoGRAMplusC4D consortium. This study mainly combined Cardiogram 1000 Genomes, the UK Biobank SOFT coronary artery disease (CAD) and two other small case-control studies in people largely of European descent (cases ≤ 76,014, controls ≤ 264,785) [32]. The inclusive CAD phenotype, i.e., SOFT CAD cases, was defined as people with fatal or nonfatal myocardial infarction, or percutaneous transluminal coronary angioplasty or coronary artery bypass grafting, or chronic IHD or angina [32]. Diagnoses were based on medical records and self-report. Genetic associations with LDL cholesterol, HDL cholesterol and triglycerides (quantile), were obtained from the UK Biobank in participants (*n* = 343,621 for LDL cholesterol, *n* = 315,133 for HDL cholesterol and *n* = 343,992 for triglycerides) based on people of white British ancestry, adjusted for the first 20 principal components, age, age^2^, sex, age × sex, and age^2^ × sex [33]. Genetic associations with apoB were obtained from GWAS summary statistics from 14 European cohorts of blood metabolites in up to 24,925 individuals [34].

### 2.3. Statistical Aanalyses

#### 2.3.1. Univariable MR Analyses

F-statistics were used to indicate instrument strength approximated by averaging the SNP specific F-statistics (approximated by the square of beta for exposure divided by its variance) [36]. We calculated the power based on the approximation that the sample size for an MR study is the sample size for exposure on outcome observationally divided by the r^2^ for genetic variants on exposure [37]. The r^2^ for the genetic variants on apoE isoforms was not given in in the KORA study (n ≤ 997), but a GWAS with a sample size of 1000 would be expected to explain 3.5% of the variance in a continuous variable with 80% power [38]. As such, here we assumed the maximum r^2^ to be 3% in the power analysis using KORA.

##### Main Analyses

We obtained univariable MR estimates using inverse variance weighting (IVW) with fixed (<4 SNPs) or multiplicative random effects (4+ SNPs) [39] to meta-analyze the SNP-specific Wald estimates. Given three isoforms were tested against one primary outcome IHD, we used a Bonferroni correction for multiple testing [40], giving a corrected P value of 0.017 (0.05/3).

##### Sensitivity Analyses

Steiger filtering was used to detect invalid genetic predictors that were potentially predictors of the outcome rather than the exposure by testing whether the approximated SNP specific r^2^ for exposure was larger than that of outcome [41]. MR-Egger was used to test directional pleiotropy assuming the InSIDE (Instrument strength independent of the direct effect) assumption, where a non-zero intercept indicates a potentially invalid IVW estimate [42]. A weighted median (WM) was used because it is robust when ≤50% of the weight comes from invalid instruments [43]. MR-PRESSO detects potentially invalid instruments (horizontal pleiotropic outliers) statistically for 4 or more independent SNPs and gives corrected estimates after removing these outliers [44]. We reported corrected estimates from MR-PRESSO where relevant.

#### 2.3.2. Multivariable MR Analyses

Given some genetic predictors predicted more than one isoform of apoE, we used multivariable MR to assess direct effects of apoE2, apoE3 and apoE4 on IHD, lipids and apoB, taking into account correlations between genetic variants based on the 1000 Genomes phase 3 data obtained from the “ld_matrix” function in the TwoSampleMR R package. To further investigate whether the effects of apoE isoforms are relevant to apoB, we also conducted multivariable analyses of apoE on IHD adjusting for apoB. Estimates in multivariable analyses were obtained using IVW or MR-Egger depending on pleiotropy assessed from the MR-Egger intercept [45,46] orientated to apoE2. We also calculated the Q-statistic for instrument pleiotropy using the WSpiller/MVMR package [47].

All statistical analyses were conducted using R version 3.6.2 (The R Foundation for Statistical Computing, Vienna, Austria). The MendelianRandomization and MRPRESSO R packages were used for the MR estimates. The MR study only uses published or publicly available data. No original data were collected for the MR study. Ethical approval for each of the studies included in the investigation can be found in the original publications (including informed consent from each participant).

## 3. Results

### 3.1. Instrument Strength and Power Calculations

The F-statistics for the genetic instruments for plasma apoE2, apoE3 and apoE4 were 29.4, 21.5 and 21.7, respectively. The study had 80% power at an α of 0.05 to detect an odds ratio of 1.07 for per inversed-normalized probe level change in each apoE isoform on IHD; power calculations for other outcomes are in Table S1. The genetic variants used and their associations with the exposures and outcomes are available in Tables S2–S4, with the correlation matrix for these SNPs in Table S5.

### 3.2. Genetically Predicted Plasma ApoE (ApoE2, ApoE3 and ApoE4) on IHD

**Figure 1:**
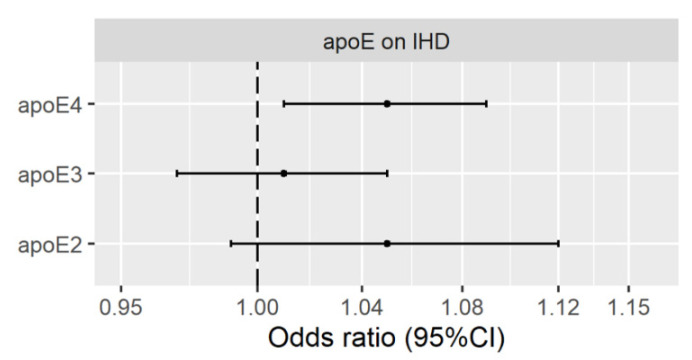
Univariable MR estimates for plasma apolipoprotein E2 (apoE2), apolipoprotein E3 (apoE3) and apolipoprotein E4 (apoE4) on ischemic heart disease (IHD) using inverse variance weighting with genetic predictors from the KORA study.

Figure 1 shows using univariable MR with SNPs from KORA, plasma apoE2 and apoE3 were not clearly associated with IHD using IVW or any other method (Table S6, Figures S1–S3), except apoE2 was nominally positively associated with IHD using WM (Table S6, Figure S2). Figure 1 also shows plasma apoE4 was positively associated with IHD after Bonferroni correction using IVW, with directionally consistent results from other methods (Table S6, Figure S4). None of the SNPs were removed after Steiger filtering (Table S2).

**Figure 2:**
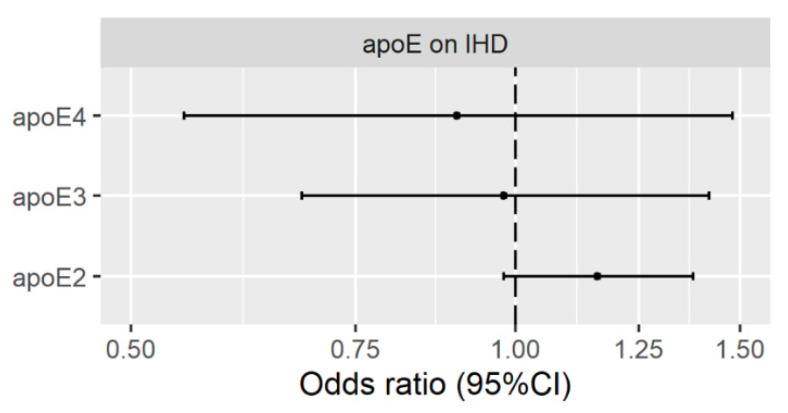
Multivariable MR estimates for plasma apolipoprotein E2 (apoE2), apolipoprotein E3 (apoE3) and apolipoprotein E4 (apoE4) on ischemic heart disease (IHD) using inverse variance weighting with genetic predictors from the KORA study.

Figure 2 shows that in multivariable MR with SNPs from KORA, apoE2 was not clearly positively associated with IHD using IVW or MR-Egger, while the associations of apoE3 and apoE4 with IHD were null. The MR-Egger intercept and Q statistic gave no indication of pleiotropy (Table S7).

In multivariable MR adjusting for apoB, the associations of apoE isoforms with IHD were generally null using IVW or MR-Egger (Table S8).

#### Validation Using Genetic Instruments from the INTERVAL Study

In the univariable analysis, both apoE2 and apoE3 were positively associated with IHD (Table S9). None of the SNPs were removed after Steiger filtering (Table S4).

### 3.3. Genetically Predicted Plasma ApoE (ApoE2, ApoE3 and ApoE4) on LDL Cholesterol, HDL Cholesterol, Triglycerides and ApoB

**Figure 3:**
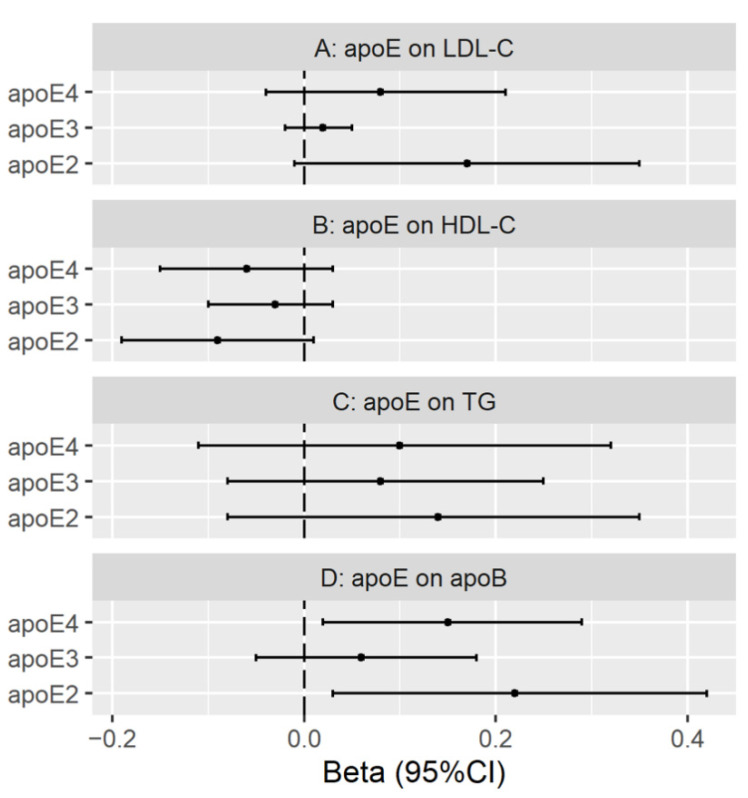
Univariable MR estimates for plasma apolipoprotein E2 (apoE2), apolipoprotein E3 (apoE3) and apolipoprotein E4 (apoE4) on low-density lipoprotein cholesterol (LDL-C), high-density lipoprotein cholesterol (HDL-C), triglycerides (TG) and apolipoprotein B (apoB) using inverse variance weighting with genetic predictors from the KORA study.

Figure 3 shows that in the univariable MR with SNPs from KORA, plasma apoE2 and apoE4 were positively associated with apoB using IVW, with directionally consistent estimates from MR-Egger (Table S10). Plasma apoE2, apoE3 and apoE4 were not clearly associated with other lipids using IVW, but apoE2 was positively associated with LDL cholesterol and triglycerides, and inversely associated with HDL cholesterol using WM (Table S10). None of the SNPs were removed after Steiger filtering (Table S2).

**Figure 4:**
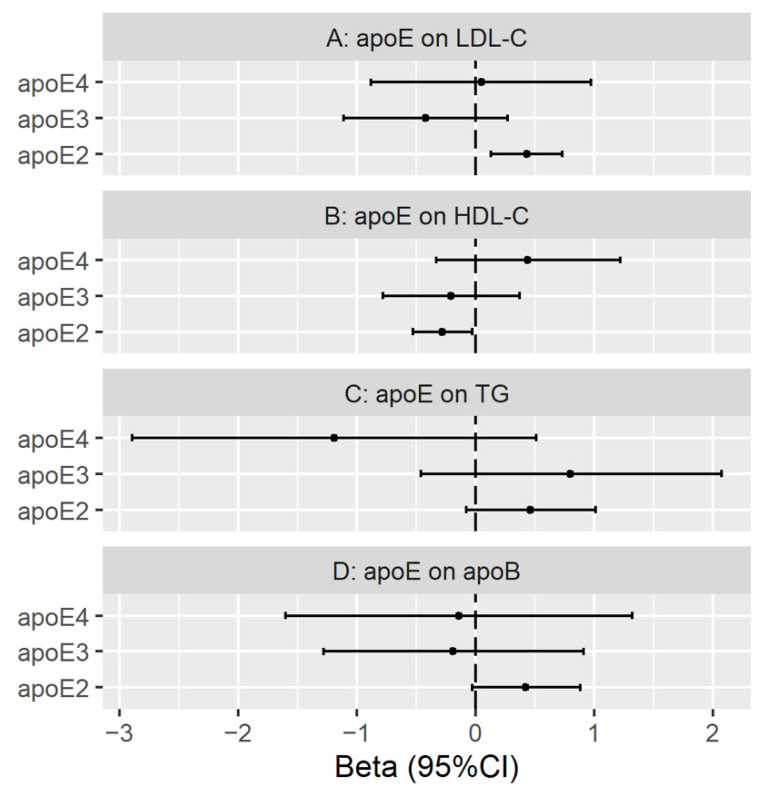
Multivariable MR estimates for plasma apolipoprotein E2 (apoE2), apolipoprotein E3 (apoE3) and apolipoprotein E4 (apoE4) on low-density lipoprotein cholesterol (LDL-C), high-density lipoprotein cholesterol (HDL-C), triglycerides (TG) and apolipoprotein B (apoB) using inverse variance weighting with genetic predictors from the KORA study.

Figure 4 shows that in multivariable MR with SNPs from KORA apoE2 was positively associated with LDL cholesterol, inversely with HDL cholesterol but not clearly with triglycerides or apoB using IVW or MR-Egger, and the effects of apoE3 and apoE4 were null, with MR-Egger intercept and Q statistic giving no indication of pleiotropy (Table S11).

#### Validation with Genetic Instruments from the INTERVAL Study

In the univariable analysis, both apoE2 and apoE3 were positively associated with LDL cholesterol and apoB, inversely with HDL cholesterol, and apoE2 was also inversely associated with triglycerides (Table S12). None of the SNPs were removed after Steiger filtering, except for rs1065853 in the association of apoE2 with apoB (Table S4), but removing this SNP gave a directionally consistent estimate.

## 4. Discussion

This study, for the first time, shows estimates of causal effects of apoE isoforms on IHD, lipid profile and apoB. These findings are somewhat consistent with previous observational studies in humans showing plasma apoE positively associated with IHD [26,48]. The findings on lipids are also consistent with previous opinions concerning the functionality of apoE isoforms, i.e., that apoE2 and apoE4 are associated with abnormal plasma lipids, while apoE3 is not [1,24]. This study adds by showing the possible harmful effects of some specific apoE isoforms, apoE2 and apoE4, on IHD, which might possibly be mediated by apoB.

To date observational studies or MR studies in humans have mainly shown the overall associations of plasma apoE with IHD rather than associations for specific isoforms. Evidence concerning specific apoE isoforms is mainly based on the characteristics of apoE protein structure and effects of *APOE* genetic variants. A previous MR study showed no effect of apoE overall on CVD mortality, which could be due to different effects of different apoE isoforms, or to considering CVD mortality [29] when effects on CVD mortality could be smaller than on IHD due to competing risk of other causes of death. From a mechanistic perspective, different isoforms of plasma apoE have different structures and possibly different effects on lipid profile [12,24], and hence on IHD. Although the biological mechanisms by which apoE might affect IHD are not completely clear, its role in lipid regulation is thought to be one of the pathways [49]. Previous studies suggest that apoE2 reduces lipid clearance, leading to increased plasma lipids [24]; while apoE4 has a higher affinity for lipids, and thus down-regulates the LDL receptor, which could lead to higher LDL cholesterol [1]. Consistent with these insights on the functionality of apoE isoforms [1,24], we found apoE2 and apoE4 increased LDL cholesterol and apoB which could drive effects on IHD, given recent evidence that apoB may also be relevant to effective lipid modification [4,5,6,7]. Most studies on *APOE* genetic variants have shown that *APOE* ε4 is positively associated with IHD [14,15,16,17,18] while an association of *APOE* ε2 with IHD is less definitive. In this study we found plasma apoE4 positively associated with IHD, apoB and possibly LDL cholesterol, consistent with the effects of the *APOE* ε4 allele, indicating plasma apoE protein might be a potential drug target for intervention, whilst an association for apoE2 cannot be excluded.

MR studies have to satisfy the three key assumptions of instrumental variable analysis, i.e., relevance, independence and exclusion restriction. First, we used genetic predictors associated at *p*-value < 10^−5^ to predict plasma apoE2, apoE3 and apoE4 in the main analyses, given the sample size of the GWAS in KORA study is small (up to 997), which tends to generate higher p-values than larger GWASs. Such selection in the main analysis could introduce invalid SNPs, but the F-statistics were >10, and one of the SNPs (rs4420638) used is a proxy of *APOE* functional variant rs429358 (r^2^ = 0.72). We also used independent predictors at genome-wide significance from a larger GWAS for apoE2 and apoE3 as a validation. Moreover, weak instrument bias is usually towards the null in separate sample MR studies [50], but we found some significant associations of apoE2 and apoE4 with IHD and apoB. Second, to check the randomization, i.e., independence, we tested the associations, at genome-wide significance, of the genetic instruments with several possible confounders including current tobacco smoking, alcohol intake frequency and walking frequency using summary statistics from the UK Biobank [33]. We did not find any SNPs used in either the main or validation analyses associated with these potential confounders. Moreover, the underlying GWASs were mainly conducted in people of European ancestry making it less likely to be confounded by population stratification. Third, to address exclusion restriction, i.e., the instrument should only be linked to the outcome only via its effect on the exposure, we used several sensitivity analyses with different assumptions including MR-Egger, WM and MR-PRESSO to detect invalid SNPs and to give corrected estimates where available. We also searched Phenoscanner to check for potential known pleiotropy (Table S13). However, these techniques to address pleiotropy use statistical techniques and observed associations when the key issue is whether apoE isoforms determine and act via LDL cholesterol and apoB (vertical pleiotropy) which does not violate the exclusion restriction assumption or whether LDL cholesterol and apoB represent pleiotropic effects of the genetic predictors of apoE (horizontal pleiotropy).

This MR study made use of large GWASs. However, several limitations are worth mentioning. First, the genetic instruments are from relatively small samples so estimates for apoE isoforms should be interpreted very cautiously. Second, we used summary statistics from two proteome GWASs but the units are possibly different, making it hard to compare the magnitude of effects. However, MR studies give estimates for lifetime exposures, so the focus is more on the direction of association as the magnitude might not correspond exactly to the effect of a time limited intervention. In this study, we used two different studies for the exposures, one as the main analysis and the other as a sensitivity analysis, in order to see whether we obtained a similar interpretation rather than to compare the magnitude of the estimates. Third, we assumed linear associations because a dose-response is seen as an indicator of a causal effect. Fourth, MR estimates largely rely on the InSIDE assumption meaning they cannot distinguish between effects of the exposure and a pre-cursor with the same genetic predictors. Fifth, physiological functions of apoE differ by isoform, here we cannot exclude the possibility that our genetic variants do not distinguish each isoform exclusively, but we conducted multivariable MR of apoE isoforms on IHD, and found apoE2 is the isoform that possibly has the main effect, although it was not statistically significant, possibly because of lack of power. Further studies using stronger genetic instruments are needed to validate this finding. Sixth, we could not exclude the possibility of reverse associations, such as LDL cholesterol or apoB affecting plasma apoE, and we were not able to assess these associations due to the relatively small sample size and number of SNPs in the apoE GWAS. Seventh, given *APOE* genetic variants are associated with longevity [51], studies of apoE on IHD could be open to selection bias due to death prior to recruitment from IHD or a competing risk of IHD [52]. As such, studies in younger people to avoid such bias are needed for further clarification. Eighth, the exposure and outcome (IHD) GWAS had some sample overlap, which could bias the estimates [53]. However, minimal bias would be expected, given the bias due to sample overlap in two sample MR studies is proportional to the percentage of overlap and the relative bias (i.e., reciprocal of the F statistics) [53], both of which were small here (percentage of overlap <0.3%; reciprocal of the F statistics: <0.05). Ninth, the instruments are from a small discovery GWAS so there could be “winners curse”. Tenth, this study largely pertains to people of European ancestry, however we would expect transportability to other populations given protein functionality is usually consistent. Finally, MR estimates give the effects of life-long exposure, which might over-estimate effects in real-world settings when considering efficacy of interventions.

## 5. Conclusions

Consistent with previous observational studies, we found apoE isoforms, i.e., apoE2 and apoE4 might be positively associated with IHD, LDL cholesterol and apoB. After adjusting for apoB, the observed associations of apoE isoforms with IHD were not evident. ApoE plays an important role in lipid regulation, one of the most important causes of IHD. As such, apoE might serve as a potential therapeutic target, potentially modulated by some lipids lowering drugs, such as statins [10,11]. The findings from this MR study raise the question as to whether apoE isoforms are potential targets of intervention for IHD prevention and treatment or are pleiotropic effects of LDL cholesterol and apoB, which requires further investigation.

## Data Availability

The datasets generated and/or analysed during the current study are available in supplementary files.

## Supplementary Materials

The following are available online at https://www.mdpi.com/article/10.3390/nu13072215/s1, Figure S1: SNP- specific estimates of the associations of plasma apolipoprotein E2 (apoE2), apolipoprotein E3 (apoE3) and apolipoprotein E4 (apoE4) with ischemic heart disease (IHD) using genetic predictors from the KORA study, Figure S2: Scatter plot of the associations (beta coefficient) of the SNPs with plasma apolipoprotein E2 (ApoE2) and ischemic heart disease (IHD) using Mendelian Randomization with different methods, Figure S3: Scatter plot of the associations (beta coefficient) of the SNPs with plasma apolipoprotein E3 (ApoE3) and ischemic heart disease (IHD) using Mendelian Randomization with different methods, Figure S4: Scatter plot of the associations (beta coefficient) of the SNPs with plasma apolipoprotein E4 (ApoE4) and ischemic heart disease (IHD) using Mendelian Randomization with different methods, Table S1: Results of power calculation, Table S2: Summary statistics and allele information of the independent genetic instruments predicting apolipoprotein E from KORA study in the univariable analyses, Table S3: Summary statistics and allele information of the genetic instruments predicting apolipoprotein E from KORA study in the multivariable analyses, Table S4: Summary statistics and allele information of the independent genetic instruments predicting apolipoprotein E from INTERVAL study, Table S5: Correlation matrix of the genetic instruments predicting apolipoprotein E from KORA study in the multivariable analyses, Table S6: Estimates of the effect of plasma apolipoprotein E (apoE) isoforms on ischemic heart disease (IHD) using genetic predictors from KORA study in univariable Mendelian Randomization analysis, Table S7: Estimates of the effect of plasma apolipoprotein E (apoE) isoforms on ischemic heart disease using genetic predictors from KORA study in multivariable Mendelian Randomization analysis, Table S8: Estimates of the direct effect of plasma apolipoprotein E (apoE) isoforms on ischemic heart disease adjusted for apolipoprotein B using genetic predictors from KORA study in multivariable Mendelian Randomization analysis, Table S9: Estimates of the effect of plasma apolipoprotein E (apoE) isoforms on ischemic heart disease (IHD) using genetic predictors from INTERVAL study in univariable Mendelian Randomization analysis, Table S10: Estimates of the effect of plasma apolipoprotein E (apoE) isoforms on low-density lipoprotein (LDL), high-density lipoprotein (HDL) cholesterol, triglycerides (TG) and apolipoprotein B (apoB) using genetic predictors from KORA study in univariable Mendelian Randomization analysis, Table S11: Estimates of the effect of plasma apolipoprotein E (apoE) isoforms on low-density lipoprotein (LDL), high-density lipoprotein (HDL) cholesterol, triglycerides (TG) and apolipoprotein B (apoB) using genetic predictors from KORA study in multivariable Mendelian Randomization analysis, Table S12: Estimates of the effect of plasma apolipoprotein E (apoE) isoforms on low-density lipoprotein (LDL), high-density lipoprotein (HDL) cholesterol, triglycerides (TG) and apolipoprotein B (apoB) using genetic predictors from INTERVAL study in univariable Mendelian Randomization analysis, Table S13: Genetic instruments predicting apoE2, apoE3 or apoE4 and potentially pleiotropic effects from Phenoscanner.
