# Supplementary material for: Investigating Effects of Plasma Apolipoprotein E on Ischemic Heart Disease Using Mendelian Randomization Study
Source: Nutrients. 2021 Jun 28;13(7):2215. doi: 10.3390/nu13072215 (PMC8308265; doi:10.3390/nu13072215)
Supplement: Supplementary file 1 [file nutrients-13-02215-s001.zip › nutrients-1201034-supplementary.pdf]

Supplementary TableS1 Results of power calculation.

Exposure	Outcome	Minimum effect size (odds ratio/ beta) with at least 80% power at 5% α to detect
ApoE	IHD	1.068
	LDL	0.028
	HDL	0.029
	TG	0.028
	ApoB	0.102

Supplementary Table S2 Summary statistics and allele information of the independent genetic instruments predicting apolipoprotein E from KORA study in the univariable analyses.

SNP	Exposure	EA	OA	Beta_Exposi	SE_Exposur	Beta_IHD	SE_IHD	P_Steiger filtering_IHD	Beta_LDL	SE_LDL	P_Steiger filt	Beta_HDL	SE_HDL	P_Steiger filt	Beta_TG	SE_TG	P_Steiger filt	Beta_APOB	SE_APOB	P_Steiger filtering_APOB
rs10129240	ApoE2	T	C	-0.208	0.0454347	0.00628	0.00866	6.74E-06	-0.000679	0.0025567	5.65E-06	-0.003236	0.0024718	7.49E-06	0.0031542	0.0025192	7.26E-06	-0.006593	0.010959	1.55E-05
rs16861605	ApoE2	A	G	-0.3316	0.0636346	0.00334	0.01227	2.51E-07	-0.003276	0.0034175	2.96E-07	-0.002638	0.0033033	2.86E-07	-0.002792	0.0033672	2.85E-07	0.004511	0.01774	6.01E-07
rs17794309	ApoE2	T	G	-0.6333	0.1334106	0.00133	0.02813	2.41E-06	0.0083491	0.0067217	3.27E-06	0.0012425	0.0064954	2.50E-06	0.0073505	0.0066218	3.16E-06	0.056326	0.036973	2.39E-05
rs4420638	ApoE2	G	A	0.4193	0.0562366	0.04936	0.0125	1.98E-12	0.16698	0.0029812	8.80E-09	-0.062834	0.0028951	7.37E-10	0.053165	0.0029492	1.48E-10	0.148882	0.012319	9.78E-06
rs6589565	ApoE2	A	G	0.3947	0.0868236	0.04432	0.01387	1.77E-05	0.053295	0.0047092	9.10E-05	-0.10898	0.0045518	0.0014808	0.26434	0.0046185	0.0037523	0.17028	0.018706	0.013926
rs10400332	ApoE3	G	A	0.2602	0.0548251	0.01448	0.01044	3.86E-06	-0.000396	0.0029208	2.46E-06	0.0044744	0.0028248	3.67E-06	-0.004381	0.0028779	3.53E-06	-0.012849	0.014983	9.81E-06
rs12123018	ApoE3	C	T	-0.2104	0.0448232	0.0074	0.00864	4.14E-06	0.0038694	0.0024334	4.59E-06	-0.000133	0.002354	3.10E-06	0.0018732	0.0023973	3.72E-06	0.008801	0.010688	1.18E-05
rs12906588	ApoE3	T	G	-0.1957	0.0437025	0.00956	0.00847	1.22E-05	0.0017166	0.0024513	1.00E-05	-0.00312	0.0023695	1.19E-05	0.0044292	0.0024152	1.33E-05	-0.010382	0.010505	3.49E-05
rs16861605	ApoE3	A	G	-0.2837	0.0637528	0.00334	0.01227	1.05E-05	-0.003276	0.0034175	1.21E-05	-0.002638	0.0033033	1.18E-05	-0.002792	0.0033672	1.18E-05	0.004511	0.01774	2.06E-05
rs2680860	ApoE3	T	C	-0.3061	0.0662124	0.00904	0.01255	5.55E-06	-0.005455	0.0037083	6.21E-06	-0.001756	0.0035843	4.88E-06	-0.000834	0.0036534	4.53E-06	0.00453	0.014337	9.42E-06
rs614035	ApoE3	C	T	0.2112	0.0469855	-0.00333	0.00861	8.87E-06	0.0011676	0.0025124	8.75E-06	-0.012969	0.0024293	3.03E-05	0.010677	0.0024754	2.24E-05	-0.00922	0.011016	2.76E-05
rs6589565	ApoE3	A	G	0.414	0.0866109	0.04432	0.01387	6.09E-06	0.053295	0.0047092	3.40E-05	-0.10898	0.0045518	0.000651	0.26434	0.0046185	0.001752	0.17028	0.018706	0.0072478
rs7443900	ApoE3	A	G	0.8284	0.1715824	0.01187	0.02513	1.90E-06	-0.013154	0.0090739	2.34E-06	0.0083872	0.0087691	2.08E-06	-0.012943	0.0089414	2.34E-06	0.057668	0.032144	1.88E-05
rs10402271	ApoE4	G	T	0.2177	0.0480044	0.01859	0.00878	1.29E-05	0.082908	0.0025169	0.003886	-0.019145	0.002437	4.70E-05	0.0012614	0.0024835	7.33E-06	0.060883	0.010696	0.001707
rs16861605	ApoE4	A	G	-0.3139	0.0634783	0.00334	0.01227	9.86E-07	-0.003276	0.0034175	1.15E-06	-0.002638	0.0033033	1.12E-06	-0.002792	0.0033672	1.11E-06	0.004511	0.01774	2.20E-06
rs16950728	ApoE4	A	G	-0.2695	0.0597296	-0.01335	0.01129	1.07E-05	-0.00619	0.0032578	1.16E-05	-0.004633	0.0031494	1.06E-05	0.0031471	0.0032098	9.21E-06	-0.012449	0.013294	2.86E-05
rs576405	ApoE4	T	C	-0.1945	0.0430977	-0.00863	0.00821	1.01E-05	0.0061314	0.0023738	1.36E-05	0.0080295	0.0022954	1.77E-05	-0.004507	0.0023389	1.16E-05	-0.018731	0.010057	6.74E-05
rs6589565	ApoE4	A	G	0.3864	0.0865398	0.04432	0.01387	2.53E-05	0.053295	0.0047092	0.000126	-0.10898	0.0045518	0.001943	0.26434	0.0046185	0.004823	0.17028	0.018706	0.017251
rs7443900	ApoE4	A	G	0.8456	0.1711048	0.01187	0.02513	1.08E-06	-0.013154	0.0090739	1.33E-06	0.0083872	0.0087691	1.18E-06	-0.012943	0.0089414	1.33E-06	0.057668	0.032144	1.14E-05

EA: effect allele, OA: other allele, ApoE: apolipoprotein E, IHD: ischemic heart disease, LDL: low desity-lipoprotein cholesterol, HDL: high density-lipoprotein cholesterol, TG: triglycerides, APOB: apolipoprotein B

Supplementary Table S3 Summary statistics and allele information of the genetic instruments predicting apolipoprotein E from KORA study in the multivariable analyses.

SNP	EA	OA	Beta_apoe2	Beta_apoe3	Beta_apoe4	SE_apoe2	SE_apoe3	SE_apoe4	Beta_IHD	SE_IHD	Beta_LDL	SE_LDL	Beta_HDL	SE_HDL	Beta_TG	SE_TG	Beta_APOB	SE_APOB
rs10129240	C	T	0.208	0.1801	0.1981	0.0454347	0.0455028	0.0453318	-0.00628	0.00866	0.000679	0.0025567	0.0032358	0.0024718	-0.003154	0.0025192	0.006593	0.010959
rs10400332	A	G	-0.215	-0.2602	-0.2302	0.0551141	0.0548251	0.0548487	-0.01448	0.01044	0.0003956	0.0029208	-0.004474	0.0028248	0.0043806	0.0028779	0.012849	0.014983
rs10402271	T	G	-0.242	-0.1803	-0.2177	0.048054	0.0482602	0.0480044	-0.01859	0.00878	-0.082908	0.0025169	0.019145	0.002437	-0.001261	0.0024835	-0.060883	0.010696
rs12123018	T	C	0.1868	0.2104	0.1968	0.0449795	0.0448232	0.0447782	-0.0074	0.00864	-0.003869	0.0024334	0.0001331	0.002354	-0.001873	0.0023973	-0.008801	0.010688
rs12906588	G	T	0.1713	0.1957	0.1854	0.0438556	0.0437025	0.0436441	-0.00956	0.00847	-0.001717	0.0024513	0.0031198	0.0023695	-0.004429	0.0024152	0.010382	0.010505
rs16861605	G	A	0.3316	0.2837	0.3139	0.0636346	0.0637528	0.0634783	-0.00334	0.01227	0.0032763	0.0034175	0.0026379	0.0033033	0.0027923	0.0033672	-0.004511	0.01774
rs16950728	G	A	0.2293	0.2552	0.2695	0.0601206	0.0599342	0.0597296	0.01335	0.01129	0.0061901	0.0032578	0.004633	0.0031494	-0.003147	0.0032098	0.012449	0.013294
rs17794309	G	T	0.6333	0.4856	0.509	0.1334106	0.1338479	0.1334557	-0.00133	0.02813	-0.008349	0.0067217	-0.001243	0.0064954	-0.007351	0.0066218	-0.056326	0.036973
rs2680860	C	T	0.2649	0.3061	0.2919	0.0665913	0.0662124	0.0661605	-0.00904	0.01255	0.0054546	0.0037083	0.0017558	0.0035843	0.000834	0.0036534	-0.00453	0.014337
rs4420638	A	G	-0.4193	-0.07585	-0.1709	0.0562366	0.0577245	0.0573875	-0.04936	0.0125	-0.16698	0.0029812	0.062834	0.0028951	-0.053165	0.0029492	-0.148882	0.012319
rs576405	C	T	0.1625	0.1802	0.1945	0.0433796	0.0432549	0.0430977	0.00863	0.00821	-0.006131	0.0023738	-0.00803	0.0022954	0.0045071	0.0023389	0.018731	0.010057
rs614035	T	C	-0.1757	-0.2112	-0.1916	0.0472185	0.0469855	0.0469608	0.00333	0.00861	-0.001168	0.0025124	0.012969	0.0024293	-0.010677	0.0024754	0.00922	0.011016
rs6589565	G	A	-0.3947	-0.414	-0.3864	0.0868236	0.0866109	0.0865398	-0.04432	0.01387	-0.053295	0.0047092	0.10898	0.0045518	-0.26434	0.0046185	-0.17028	0.018706
rs7443900	G	A	-0.7425	-0.8284	-0.8456	0.1722338	0.1715824	0.1711048	-0.01187	0.02513	0.013154	0.0090739	-0.008387	0.0087691	0.012943	0.0089414	-0.057668	0.032144

EA: effect allele, OA: other allele, ApoE: apolipoprotein E, IHD: ischemic heart disease, LDL: low desity-lipoprotein cholesterol, HDL: high density-lipoprotein cholesterol, TG: triglycerides, APOB: apolipoprotein B

Supplementary Table S4 Summary statistics and allele information of the independent genetic instruments predicting apolipoprotein E from INTERVAL study.

SNP	Exposure	EA	OA	Beta_Exposı	SE_Exposur	Beta_IHD	SE_IHD	P_Steiger filt	Beta_LDL	SE_LDL	P_Steiger filt	Beta_HDL	SE_HDL	P_Steiger filt	Beta_TG	SE_TG	P_Steiger filt	Beta_APOB	SE_APOB	P_Steiger filtering_APOB
rs1065853	ApoE2	T	G	-0.3402	0.0461	-0.1432	0.01589	1.95E-08	-0.4724	0.0042591	1.08E-05	0.067795	0.0041813	1.23E-08	0.10931	0.0042663	1.22E-06	-0.427553	0.025979	0.292288452
rs814573	ApoE2	A	G	-0.2472	0.0316	-0.04936	0.0125	1.69E-14	-0.16698	0.0029812	1.27E-06	0.062834	0.0028951	2.40E-08	-0.053165	0.0029492	1.58E-09	-0.148882	0.012319	0.003723535
rs10401176	ApoE3	T	C	-0.2227	0.0388	-0.05207	0.01297	2.47E-07	-0.14415	0.0035688	0.0055151	0.024407	0.0034546	5.68E-07	0.022623	0.0035243	3.44E-07	-0.108809	0.017476	0.00545747
rs7412	ApoE3	T	C	-0.7217	0.0448	-0.1432	0.01589	3.06E-50	-0.4724	0.0042591	8.90E-40	0.067795	0.0041813	1.40E-47	0.10931	0.0042663	1.99E-42	-0.427553	0.025979	5.40E-14
rs814573	ApoE3	A	G	-0.2279	0.0317	-0.04936	0.0125	5.77E-11	-0.16698	0.0029812	2.41E-05	0.062834	0.0028951	7.05E-07	-0.053165	0.0029492	6.14E-08	-0.148882	0.012319	0.064252248
rs56394238	ApoE3	A	G	-0.1435	0.0249	-0.02659	0.00873	9.51E-08	-0.074942	0.0023752	0.0050709	0.015437	0.0023002	4.08E-07	0.015083	0.0023434	3.00E-07	-0.042509	0.010847	0.000189798
rs59325138	ApoE3	T	C	0.1728	0.0254	0.00115	0.00865	1.32E-11	0.061966	0.0024095	1.88E-05	0.0060019	0.0023322	7.03E-11	0.0038337	0.0023761	3.41E-11	0.034874	0.010491	5.12E-07

EA: effect allele, OA: other allele, ApoE: apolipoprotein E, IHD: ischemic heart disease, LDL: low desity-lipoprotein cholesterol, HDL: high density-lipoprotein cholesterol, TG: triglycerides, APOB: apolipoprotein B

Supplementary Table S5 Correlation matrix of the genetic instruments predicting apolipoprotein E from KORA study in the multivariable analyses.

	rs10129240_	rs16861605_	rs17794309_	rs4420638_(	rs6589565_/	rs10400332_	rs12123018_	rs12906588_	rs2680860_'	rs614035_C	rs7443900_/	rs10402271_	rs16950728_	rs576405_T_C
rs10129240_	1	-0.027146	0.0173255	-0.053502	-0.029136	0.0035879	-0.044397	0.0208294	-0.01654	0.0151947	0.040024	0.0196708	0.0435436	-0.000507
rs16861605_	-0.027146	1	0.0741729	0.0103736	0.0114679	0.0571382	0.0110766	0.0391097	0.0572151	0.0077194	0.0146071	0.0425219	0.0183742	0.0260666
rs17794309_	0.0173255	0.0741729	1	0.0044276	-0.055571	0.0877829	-0.020468	0.0514525	0.0302948	-0.028633	0.0543977	0.0222676	-0.04515	-0.008178
rs4420638_(	-0.053502	0.0103736	0.0044276	1	-0.021544	0.0162151	-0.014482	-0.0175	0.0281421	0.0188893	0.0579939	0.156756	0.0093977	-0.01606
rs6589565_/	-0.029136	0.0114679	-0.055571	-0.021544	1	-0.014391	-0.07017	0.0466928	0.030891	-0.035179	-0.032082	0.0103254	-0.032938	0.0158098
rs10400332_	0.0035879	0.0571382	0.0877829	0.0162151	-0.014391	1	-0.11201	-0.007441	0.0091956	-0.005591	0.0581069	-0.024226	-0.066219	-0.062834
rs12123018_	-0.044397	0.0110766	-0.020468	-0.014482	-0.07017	-0.11201	1	0.0645299	-0.052206	-0.029792	-0.080756	0.0210462	-0.038714	0.0682144
rs12906588_	0.0208294	0.0391097	0.0514525	-0.0175	0.0466928	-0.007441	0.0645299	1	0.0259021	-0.087028	-0.033635	0.0901275	0.0236988	-0.046614
rs2680860_'	-0.01654	0.0572151	0.0302948	0.0281421	0.030891	0.0091956	-0.052206	0.0259021	1	-0.004	0.0268699	0.0132356	0.0337507	-0.003202
rs614035_C	0.0151947	0.0077194	-0.028633	0.0188893	-0.035179	-0.005591	-0.029792	-0.087028	-0.004	1	-0.021837	-0.023397	-0.040805	0.0632833
rs7443900_/	0.040024	0.0146071	0.0543977	0.0579939	-0.032082	0.0581069	-0.080756	-0.033635	0.0268699	-0.021837	1	-0.036265	-0.0305	0.0132031
rs10402271_	0.0196708	0.0425219	0.0222676	0.156756	0.0103254	-0.024226	0.0210462	0.0901275	0.0132356	-0.023397	-0.036265	1	0.0037241	0.0365406
rs16950728_	0.0435436	0.0183742	-0.04515	0.0093977	-0.032938	-0.066219	-0.038714	0.0236988	0.0337507	-0.040805	-0.0305	0.0037241	1	-0.00109
rs576405_T_	-0.000507	0.0260666	-0.008178	-0.01606	0.0158098	-0.062834	0.0682144	-0.046614	-0.003202	0.0632833	0.0132031	0.0365406	-0.00109	1

Supplementary Table S6 Estimates of the effect of plasma apolipoprotein E (apoE) isoforms on ischemic heart disease (IHD) using genetic predictors from KORA study in univariable Mendelian Randomization analysis.

Exposure	Outcome	SNPs	Method	OR	95%CI	P value	Q statistic (p value)	MR-Egger Intercept p value	I^2_{Gx}
ApoE2	IHD	5	IVW	1.05	0.99, 1.12	0.11	16.0 (<0.01)		
			MR-Egger	1.16	0.95, 1.42	0.15		0.32	0%
			WM	1.05	1.01 ,1.10	0.02			
ApoE3		8	IVW	1.01	0.97, 1.05	0.54	14.3 (0.05)		
			MR-Egger	1.08	0.98, 1.18	0.11		0.15	0%
			WM	0.99	0.96 ,1.03	0.71			
			MR-PRESSO	0.99	0.96, 1.03	0.63			
ApoE4		6	IVW	1.05	1.01, 1.09	0.01	7.9 (0.16)		
			MR-Egger	1.02	0.93, 1.12	0.69		0.54	0%
			WM	1.05	1.00, 1.09	0.04			

Supplementary Table S7 Estimates of the effect of plasma apolipoprotein E (apoE) isoforms on ischemic heart disease using genetic predictors from KORA study in multivariable Mendelian Randomization analysis.

Exposure	SNPs	Method	OR	95%CI	P value	Q statistic (p value)	MR-Egger Intercept p value
ApoE2	14	IVW	1.16	0.98, 1.38	0.08	11.23 (0.34)	0.50
		MR-Egger	1.17	0.98, 1.40	0.08		
ApoE3		IVW	0.98	0.68, 1.42	0.91		
		MR-Egger	0.98	0.67, 1.43	0.92		
ApoE4		IVW	0.90	0.55, 1.48	0.68		
		MR-Egger	0.92	0.55, 1.53	0.74		

Supplementary Table S8 Estimates of the direct effect of plasma apolipoprotein E (apoE) isoforms on ischemic heart disease adjusted for apolipoprotein B using genetic predictors from KORA study in multivariable Mendelian Randomization analysis.

Exposure	SNPs	Method	OR	95%CI	P value	Q statistic (p value)	MR-Egger Intercept p value
ApoE2	5	IVW	1.00	0.95, 1.04	0.86	1.36 (0.51)	0.26
		MR-Egger	1.06	0.94, 1.18	0.33		
ApoE3	8	IVW	0.99	0.97, 1.02	0.72	5.03 (0.41)	0.52
		MR-Egger	1.02	0.94, 1.11	0.63		
ApoE4	6	IVW	1.02	0.94, 1.10	0.66	13.76 (0.003)	0.45
		MR-Egger	0.97	0.83, 1.13	0.68		

Supplementary Table S9 Estimates of the effect of plasma apolipoprotein E (apoE) isoforms on ischemic heart disease (IHD) using genetic predictors from INTERVAL study in univariable Mendelian Randomization analysis.

Exposure	Outcome	SNPs	Method	OR	95%CI	P value	Q statistic (p value)	MR-Egger Intercept p value	I^2_{Gx}
ApoE2	IHD	2[1]	IVW	1.38	1.29, 1.47	<0.001	10.3 ((<0.01)	-	-
ApoE3		5[1]	IVW	1.20	1.12, 1.28	<0.001	14.0 (0.01)		
			MR-Egger	1.25	1.12, 1.40	<0.001		0.36	95%
			WM	1.22	1.17, 1.28	<0.001			

[1] The outcome information of rs814573 was replaced by that of rs4420638 (r^2=0.88), and the outcome information of rs1065853 was replaced by that of rs7412 (r^2=1.0)

Supplementary Table S10 Estimates of the effect of plasma apolipoprotein E (apoE) isoforms on low-density lipoprotein (LDL), high-density lipoprotein (HDL) cholesterol, triglycerides (TG) and apolipoprotein B (apoB) using genetic predictors from KORA study in univariable Mendelian Randomization analysis.

Exposure	Outcome	SNPs	Method	Beta	95%CI	P value	Q statistic (p value)	MR-Egger Intercept p value	I^2_{Gx}
ApoE2	LDL	5	IVW	0.17	-0.01, 0.35	0.06	1762.3 (<0.01)		
			MR-Egger	0.32	-0.31, 0.94	0.32		0.63	39%
			WM	0.10	0.04, 0.15	<0.01			
			MR-PRESSO	0.14	-	-			
ApoE3		8	IVW	0.02	-0.02, 0.05	0.38	122.8 (<0.01)		
			MR-Egger	0.06	-0.05, 0.16	0.30		0.42	0%
			WM	0.00	-0.01, 0.01	0.81			
			MR-PRESSO	0.00	-0.01, 0.02	0.35			
ApoE4		6	IVW	0.08	-0.04, 0.21	0.20	925.4 (<0.01)		
			MR-Egger	-0.04	-0.40, 0.32	0.82		0.47	0%
			WM	0.02	0.00, 0.03	0.10			
ApoE2	HDL	5	IVW	-0.09	-0.19, 0.01	0.08	584.7 (<0.01)		
			MR-Egger	-0.18	-0.53, 0.16	0.30		0.58	38%
			WM	-0.06	-0.08, -0.03	<0.01			
ApoE3		8	IVW	-0.03	-0.10, 0.03	0.32	531.9 (<0.01)		
			MR-Egger	-0.10	-0.31, 0.11	0.35		0.51	0%
			WM	0.01	0.00, 0.02	0.18			
			MR-PRESSO	-0.03	-0.42, 0.36	0.52			
ApoE4		6	IVW	-0.06	-0.15, 0.03	0.21	494.9 (<0.01)		
			MR-Egger	-0.05	-0.32, 0.22	0.70		0.97	0%
			WM	-0.01	-0.02 ,0.01	0.25			
			MR-PRESSO	-0.07	-0.36, 0.23	0.22			
ApoE2	TG	5	IVW	0.14	-0.08, 0.35	0.21	2595.0 (<0.01)		
			MR-Egger	0.24	-0.53, 1.00	0.55		0.79	38%
			WM	0.05	0.03, 0.07	<0.01			
			MR-PRESSO	0.13	-	-			
ApoE3		8	IVW	0.08	-0.08, 0.25	0.31	2873.6 (<0.01)		
			MR-Egger	0.28	-0.21, 0.77	0.26		0.40	0%
			WM	0.00	-0.02, 0.01	0.51			
			MR-PRESSO	0.05	-	-			
ApoE4		6	IVW	0.10	-0.11, 0.32	0.34	2775.0 (<0.01)		
			MR-Egger	0.22	-0.41, 0.86	0.49		0.70	0%
			WM	0.01	-0.01, 0.02	0.31			
ApoE2	ApoB	5	IVW	0.22	0.03, 0.42	0.02	100.8 (<0.01)		
			MR-Egger	0.39	-0.29, 1.08	0.26		0.61	60%
			WM	0.24	0.11 ,0.36	<0.01			
ApoE3		8	IVW	0.06	-0.05, 0.18	0.27	76.1 (<0.01)		
			MR-Egger	0.23	-0.06, 0.52	0.12		0.22	0%
			WM	0.00	-0.05 ,0.05	0.97			
			MR-PRESSO	0.00	-0.05, 0.05	0.91			
ApoE4		6	IVW	0.15	0.02, 0.29	0.03	61.6 (<0.01)		
			MR-Egger	0.12	-0.24, 0.48	0.51		0.85	0%
			WM	0.08	0.01, 0.15	0.02			
			MR-PRESSO	0.12	-0.05, 0.28	0.11			

Supplementary Table S11 Estimates of the effect of plasma apolipoprotein E (apoE) isoforms on low-density lipoprotein (LDL), high-density lipoprotein (HDL) cholesterol, triglycerides (TG) and apolipoprotein B (apoB) using genetic predictors from KORA study in multivariable Mendelian Randomization analysis.

Exposure	Outcome	SNPs	Method	Beta	95%CI	P value	Q statistic (p value)	MR-Egger Intercept p value
ApoE2	LDL	14	IVW	0.43	0.13, 0.73	<0.01	5.62 (0.85)	0.45
			MR-Egger	0.42	0.11, 0.72	0.01		
ApoE3			IVW	-0.42	-1.11, 0.27	0.23		
			MR-Egger	-0.41	-1.12, 0.29	0.25		
ApoE4			IVW	0.05	-0.88, 0.97	0.92		
			MR-Egger	-0.01	-0.97, 0.94	0.98		
ApoE2	HDL	14	IVW	-0.28	-0.53, -0.03	0.03	5.02 (0.89)	0.95
			MR-Egger	-0.28	-0.54, -0.02	0.03		
ApoE3			IVW	-0.21	-0.78, 0.37	0.48		
			MR-Egger	-0.21	-0.81, 0.40	0.51		
ApoE4			IVW	0.44	-0.33, 1.22	0.26		
			MR-Egger	0.44	-0.38, 1.26	0.30		
ApoE2	TG	14	IVW	0.46	-0.08, 1.01	0.10	3.64 (0.96)	0.53
			MR-Egger	0.48	-0.09, 1.04	0.10		
ApoE3			IVW	0.80	-0.46, 2.07	0.21		
			MR-Egger	0.79	-0.52, 2.10	0.24		
ApoE4			IVW	-1.19	-2.89, 0.51	0.17		
			MR-Egger	-1.09	-2.87, 0.68	0.23		
ApoE2	ApoB	14	IVW	0.42	-0.03, 0.88	0.07	18.35 (0.05)	0.72
			MR-Egger	0.44	-0.05, 0.92	0.08		
ApoE3			IVW	-0.19	-1.28, 0.91	0.74		
			MR-Egger	-0.19	-1.33, 0.95	0.74		
ApoE4			IVW	-0.14	-1.60, 1.32	0.85		
			MR-Egger	-0.10	-1.63, 1.42	0.89		

Supplementary Table S12 Estimates of the effect of plasma apolipoprotein E (apoE) isoforms on low-density lipoprotein (LDL), high-density lipoprotein (HDL) cholesterol, triglycerides (TG) and apolipoprotein B (apoB) using genetic predictors from INTERVAL study in univariable Mendelian Randomization analysis

Exposure	Outcome	SNPs	Method	Beta	95%CI	P value	Q statistic (p value)	MR-Egger	
								Intercept p value	I^2_{Gx}
ApoE2	LDL	2[1]	IVW	1.02	1.00,1.04	<0.001	1682.9 (<0.01)	-	-
ApoE3		5[1]	IVW	0.62	0.52, 0.72	<0.001	497.7 (<0.01)		
			MR-Egger	0.71	0.54, 0.87	<0.001		0.21	95%
			WM	0.65	0.58 ,0.73	<0.001			
			MR-PRESSO	0.65	0.56, 0.74				
ApoE2	HDL	2[1]	IVW	-0.23	-0.24, -0.21	<0.001	10.4 (<0.01)	-	-
ApoE3		5[1]	IVW	-0.10	-0.18, -0.03	0.01	291.5 (<0.01)		
			MR-Egger	-0.11	-0.26, 0.05	0.18		0.99	95%
			WM	-0.10	-0.11 ,-0.08	<0.001			
			MR-PRESSO	-0.10	-0.12, -0.08	<0.001			
ApoE2	TG	2[1]	IVW	-0.04	-0.06, -0.02	<0.001	960.3 (<0.01)	-	-
ApoE3		5[1]	IVW	-0.08	-0.20, 0.05	0.24	790.9 (<0.01)		
			MR-Egger	-0.18	-0.39, 0.03	0.10		0.25	95%
			WM	-0.12	-0.15, -0.10	<0.001			
			MR-PRESSO	-0.10	-0.13, -0.08	0.01			
ApoE2	ApoB	2[2]	IVW	0.80	0.72, 0.88	<0.001	51.5 (<0.01)		
ApoE3		5[2]	IVW	0.50	0.34, 0.66	<0.001	46.0 (<0.01)		
			MR-Egger	0.70	0.44, 0.96	<0.001		0.08	90%
			WM	0.56	0.47, 0.66	<0.001			
			MR-PRESSO	0.56	0.36, 0.78	0.003			

[1] The outcome information of rs814573 was replaced by that of rs4420638 (r^2=0.88).

2 The outcome information of rs814573 was replaced by that of rs4420638 (r^2=0.88), and the outcome information of rs1065853 was replaced by that of rs7412 (r^2=1.0)

Supplementary table S13 Genetic instruments predicting apoE2, apoE3 or apoE4 and potentially pleiotropic effects from Phenoscanner

Exposure	SNP	Phenotypes
ApoE2	rs10129240	-
	rs16861605	-
	rs17794309	-
	rs4420638	Platelet count, Plateletcrit, Red cell distribution width, Coronary artery disease, Myocardial infarction, Type II diabetes, Body mass index, High density lipoprotein, Low density lipoprotein, Total cholesterol, Triglycerides, apolipoprotein B, Alzheimers disease, C reactive protein, HDL cholesterol, LDL cholesterol, LDL cholesterol response to statins, Lipoprotein associated phospholipase A2 activity Lp2, Metabolic syndrome domains Atherogenic Dyslipidemia, Metabolic syndrome domains Vascular Inflammation, Rate of age related cognitive decline, Arm fat mass left, Arm fat mass right, Arm fat percentage left, Arm fat percentage right, Body fat percentage, Forced expiratory volume in 1-second, Forced vital capacity, Illnesses of father: alzheimers disease or dementia, Illnesses of mother: alzheimers disease or dementia, Illnesses of mother: diabetes, Illnesses of siblings: alzheimers disease or dementia, Leg fat mass left, Leg fat mass right, Leg fat percentage left, Leg fat percentage right, Medication for cholesterol, blood pressure or diabetes: cholesterol lowering medication, Pulse rate, Treatment with atorvastatin, Treatment with ezetimibe, Treatment with lipitor 10mg tablet, Treatment with rosuvastatin, Treatment with simvastatin, Trunk fat mass, Trunk fat percentage, Waist circumference, Weight, Whole body fat mass, Posterior cortical atrophy, 1 alkyl 2 acetylglycerophosphocholine esterase, Alzheimer disease, Psychomotor performance
	rs6589565	Mean corpuscular hemoglobin concentration, Platelet count, Platelet distribution width, High density lipoprotein, Total cholesterol, Triglycerides, apolipoprotein B, HDL cholesterol, LDL cholesterol, Medication for cholesterol, blood pressure or diabetes: cholesterol lowering medication, Self-reported high cholesterol, Treatment with atorvastatin, Treatment with cholesterol lowering medication, Treatment with fenofibrate, Treatment with rosuvastatin, Treatment with simvastatin
ApoE3	rs10400332	-
	rs12123018	-
	rs12906588	-
	rs16861605	-
	rs2680860	-
	rs614035	-
	rs6589565	Mean corpuscular hemoglobin concentration, Platelet count, Platelet distribution width, High density lipoprotein, Total cholesterol, Triglycerides, apolipoprotein B, HDL cholesterol, LDL cholesterol, Medication for cholesterol, blood pressure or diabetes: cholesterol lowering medication, Self-reported high cholesterol, Treatment with atorvastatin, Treatment with cholesterol lowering medication, Treatment with fenofibrate, Treatment with rosuvastatin, Treatment with simvastatin
	rs7443900	-
ApoE4	rs10402271	High density lipoprotein, Low density lipoprotein, Total cholesterol, apolipoprotein B, Alzheimers disease, LDL cholesterol, Illnesses of father: alzheimers disease or dementia, Illnesses of mother: alzheimers disease or dementia, Medication for cholesterol, blood pressure or diabetes: cholesterol lowering medication, Self-reported high cholesterol, Treatment with simvastatin
	rs16861605	-
	rs16950728	-
	rs576405	-
	rs6589565	Mean corpuscular hemoglobin concentration, Platelet count, Platelet distribution width, High density lipoprotein, Total cholesterol, Triglycerides, apolipoprotein B, HDL cholesterol, LDL cholesterol, Medication for cholesterol, blood pressure or diabetes: cholesterol lowering medication, Self-reported high cholesterol, Treatment with atorvastatin, Treatment with cholesterol lowering medication, Treatment with fenofibrate, Treatment with rosuvastatin, Treatment with simvastatin
	rs7443900	-

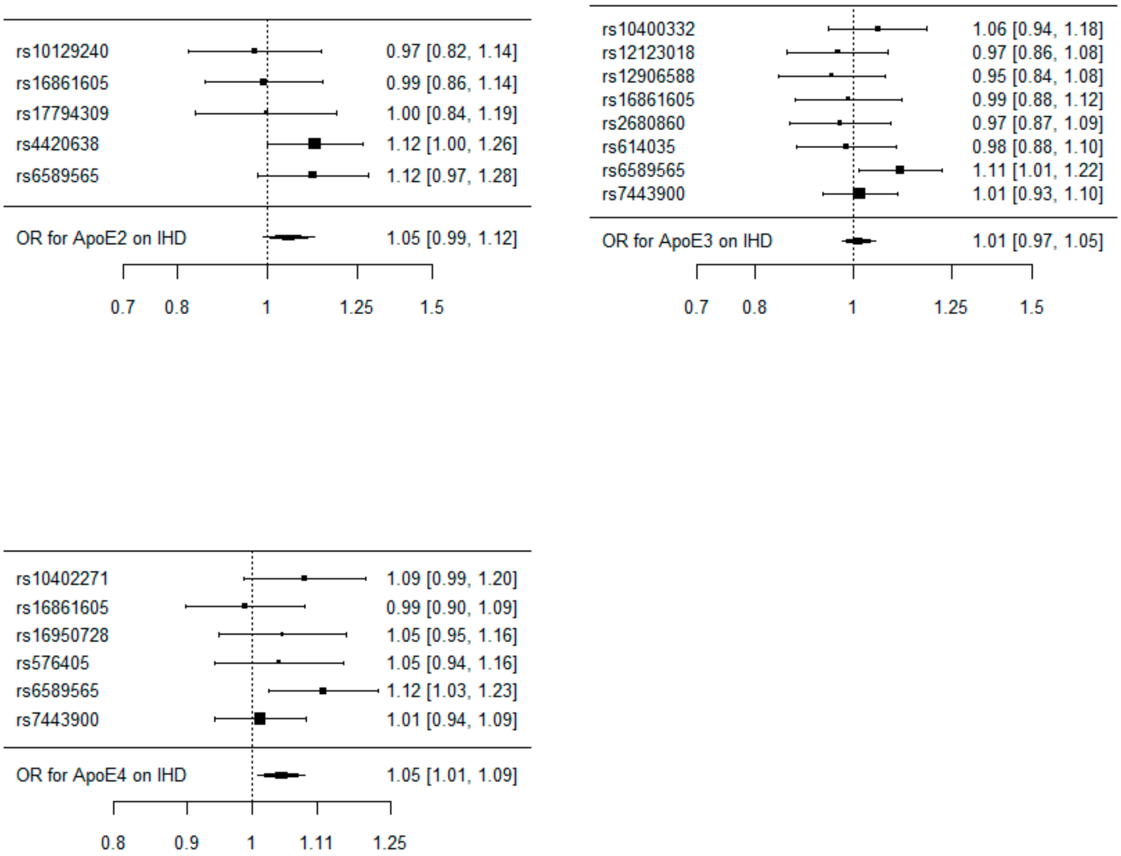

Supplementary figure 1: SNP- specific estimates of the associations of plasma apolipoprotein E2 (apoE2), apolipoprotein E3 (apoE3) and apolipoprotein E4 (apoE4) with ischemic heart disease (IHD) using genetic predictors from the KORA study.

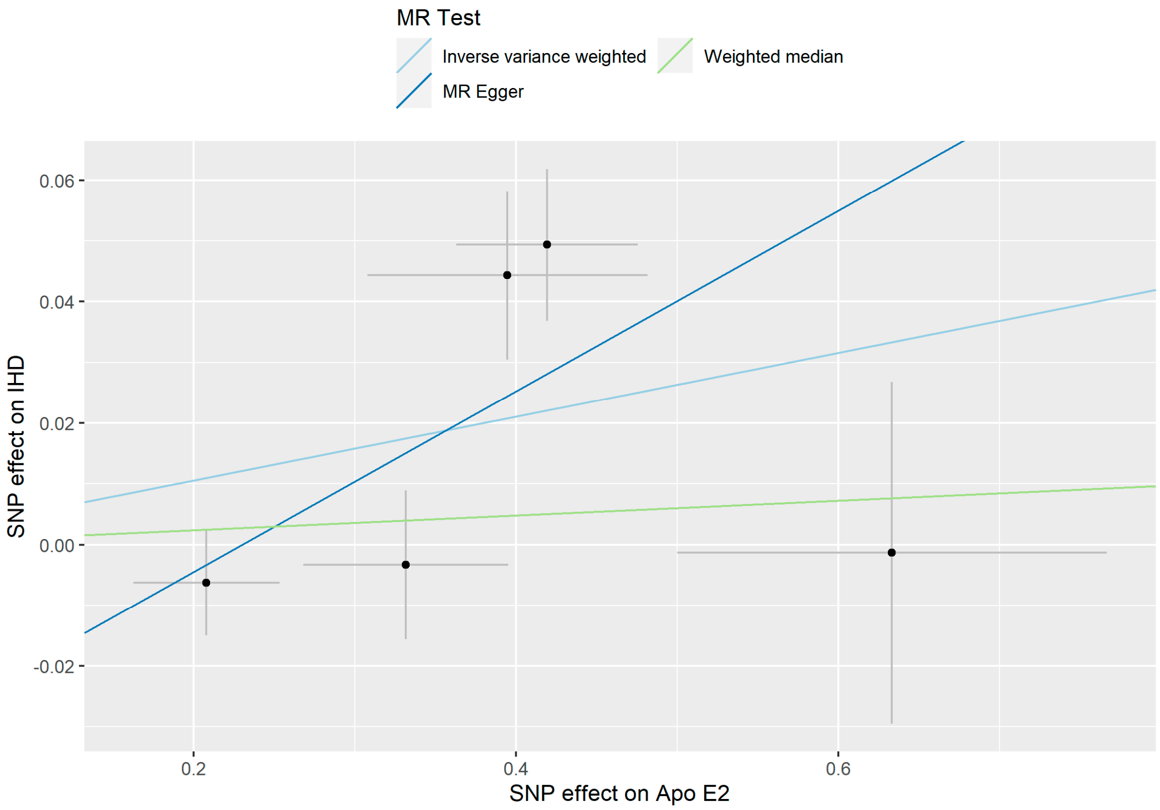

Supplementary figure 2 Scatter plot of the associations (beta coefficient) of the SNPs with plasma apolipoprotein E2 (ApoE2) and ischemic heart disease (IHD) using Mendelian Randomization with different methods.

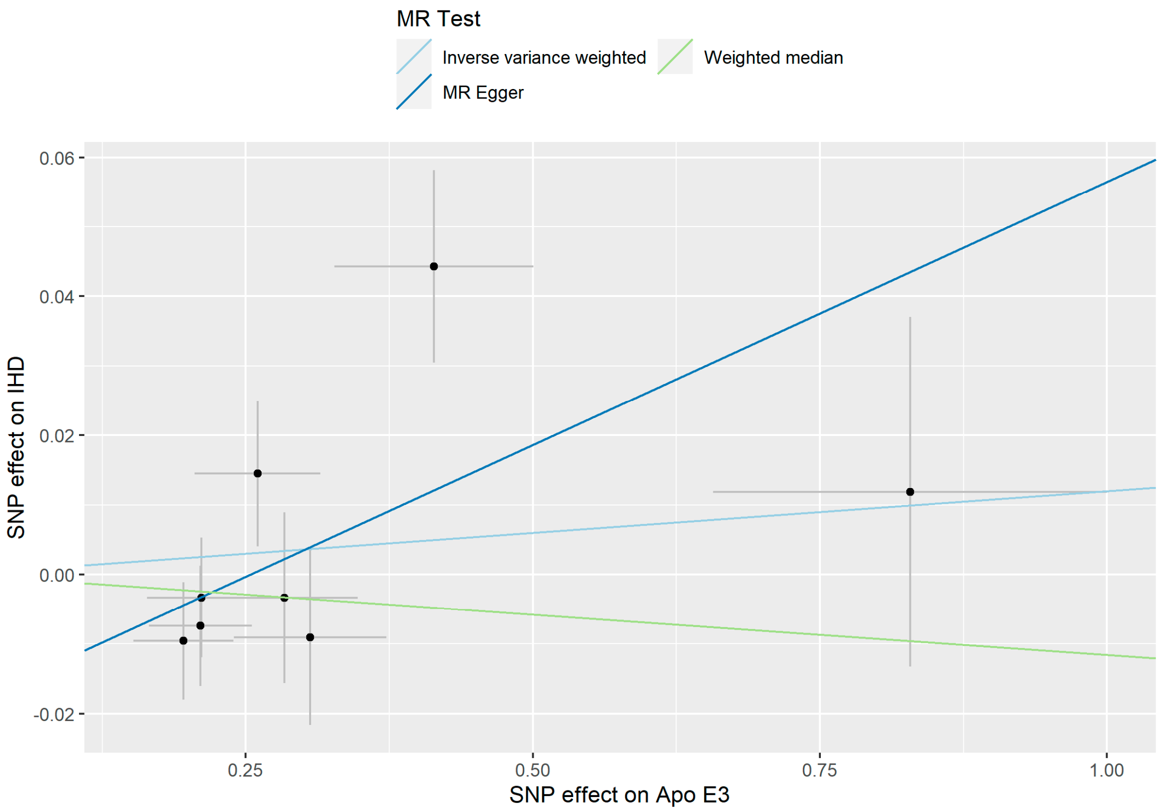

Supplementary figure 3 Scatter plot of the associations (beta coefficient) of the SNPs with plasma apolipoprotein E3 (ApoE3) and ischemic heart disease (IHD) using Mendelian Randomization with different methods.

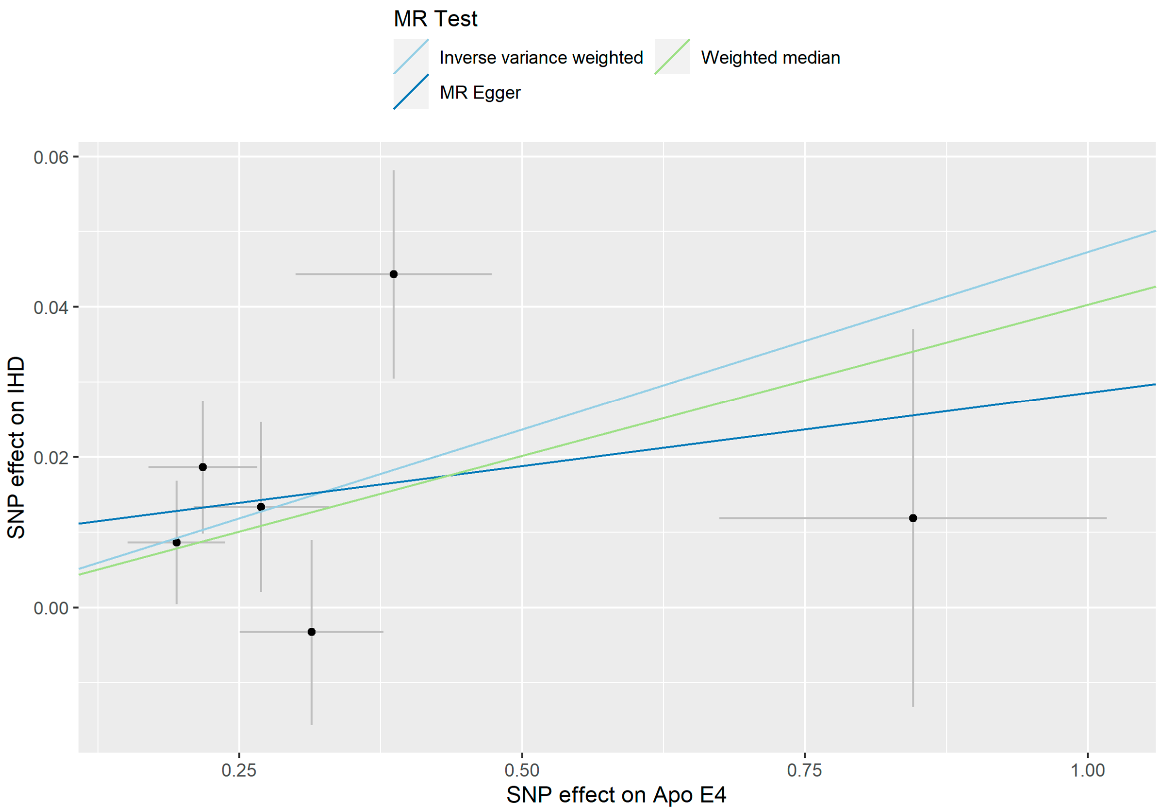

Supplementary figure 4 Scatter plot of the associations (beta coefficient) of the SNPs with plasma apolipoprotein E4 (ApoE4) and ischemic heart disease (IHD) using Mendelian Randomization with different methods
